# Desmin-p.L112Q Disturbs Filament Formation and Is a Likely-Pathogenic Variant Associated with Dilated Cardiomyopathy

**DOI:** 10.3390/jcdd13010003

**Published:** 2025-12-20

**Authors:** Alexander Lütkemeyer, Sabrina Voß, Jonas Reckmann, Joline Groß, Anna Gärtner, Jan Gummert, Hendrik Milting, Andreas Brodehl

**Affiliations:** 1 Clinic for Thoracic and Cardiovascular Surgery, Erich and Hanna Klessmann Institute, Heart and Diabetes Center North Rhine Westphalia, Ruhr-University Bochum, Georgstrasse 11, 32545 Bad Oeynhausen, Germany; alexander.luetkemeyer@uni-bielefeld.de (A.L.); svoss@hdz-nrw.de (S.V.); jonas.reckmann@uni-bielefeld.de (J.R.); jgross@hdz-nrw.de (J.G.); agaertner@hdz-nrw.de (A.G.); jgummert@hdz-nrw.de (J.G.); hmilting@hdz-nrw.de (H.M.); 2 Medical School OWL, Heart and Diabetes Center North Rhine Westphalia, Bielefeld University, Georgstrasse 11, 32545 Bad Oeynhausen, Germany

**Keywords:** desmin, dilated cardiomyopathy, desmosomes, cardiovascular genetics, intermediate filaments, mutations

## Abstract

*DES* encodes the muscle-specific intermediate filament protein desmin, which is highly relevant to the structural integrity of cardiomyocytes. Mutations in this gene cause different cardiomyopathies including dilated cardiomyopathy. Here, we functionally validate *DES*-p.L112Q using SW-13, H9c2 cells, and cardiomyocytes derived from induced pluripotent stem cells by confocal microscopy in combination with deconvolution analysis. These experiments reveal an aberrant cytoplasmic aggregation of mutant desmin. In conclusion, these functional analyses support the re-classification of *DES*-p.L112Q as a likely pathogenic variant leading to dilated cardiomyopathy.

## 1. Introduction

We have read with great interest the manuscript ‘Genetic Profiling and Phenotype Spectrum in a Chinese Cohort of Pediatric Cardiomyopathy Patients’ [1]. The authors have genetically characterized a cohort of 55 pediatric Chinese patients with dilated, hypertrophic, restrictive, and arrhythmogenic right ventricular cardiomyopathy [1]. One patient with dilated cardiomyopathy (DCM) diagnosed at the age of 13 carried a heterozygous de novo variant *DES*-p.L112Q (c.335T>A) [1], which is localized in a conserved hotspot region within the 1A domain of desmin [2]. The *DES* gene (OMIM, *125660) encodes the muscle-specific intermediate filament protein desmin [3], which consists of a highly conserved rod domain flanked by non-helical head and tail domains [4]. The rod domain is sub-divided into coil-1A, -1B-, and -2. Desmin filaments connect different cellular substructures like the cardiac desmosomes, costameres, and Z-disc [5,6,7]. Therefore, desmin is highly relevant to the structural integrity of cardiomyocytes [8].

It is known, that several pathogenic desmin genetic variants localized in close proximity to *DES*-p.L112Q disturb filament assembly, leading to aberrant cytoplasmic desmin aggregates [9,10,11,12,13,14,15]. Since the authors have not reported any functional data about *DES*-p.L112Q, we address here whether desmin filament formation is affected by this novel rare variant.

## 2. Materials and Methods

### 2.1. Plasmid Generation

The pEYFP-N1-DES plasmid has been previously described [12]. The variant *DES*-p.L112Q was inserted into this expression plasmid using the Q5 Site Directed Mutagenesis Kit (New England Biolabs, Ipswich, MA, USA) in combination with the two oligonucleotides 5′-GAAGGTGGAGCAGCAGGAGCTCAATG-3′ and 5′ TCGTTGGTGCGCGTGGTCAG-3′ (Microsynth, Balgach, Switzerland). Plasmids were prepared using the GeneJET Miniprep Kit according to the manufacturer’s instructions (Thermo Fisher, Waltham, MA, USA) and were verified by Sanger sequencing (Macrogen, Amsterdam, The Netherlands).

### 2.2. Cell Culture and Transfection

SW-13 and H9c2 cells were cultured in Dulbecco’s Modified Eagle Medium (DMEM) supplemented with 10% fetal calf serum under standard conditions (37 °C, 5% CO_2_). Induced pluripotent stem cells (iPSCs, NP00040-8, UKKi011-A, https://ebisc.org/UKKi011-A/ accessed on 5 December 2025) were kindly provided by Dr. Tomo Šarić (University of Cologne, Cologne, Germany). The iPSCs were cultured in Essential 8 Medium (Thermo Fisher Scientific, Waltham, MA, USA) on vitronectin-coated cell culture plates. One day before transfection, the cells were transferred to µ-Slide 8-Well chambers (ibidi, Gräfelfing, Germany). Transfections were performed using Lipofectamin 3000 according to the manufacturer’s instructions (Thermo Fisher).

### 2.3. Differentiation of Induced Pluripotent Stem Cells into Cardiomyocytes

iPSCs were differentiated into cardiomyocytes by modulation of the Wnt-pathway as previously described in detail [16]. For metabolic selection, iPSC-derived cardiomyocytes were selected with glucose-free RPMI 1640 Medium (Thermo Fisher Scientific) supplemented with 4 mM sodium-lactate for five days. iPSC-derived cardiomyocytes were cultured for maturation more than 100 days in cardio culture medium as previously described [16].

### 2.4. Fixation and Staining

Cells were washed twice with phosphate-buffered saline (PBS, Thermo Fisher Scientific). Afterwards, the cells were fixated with 4% Histofix (Carl Roth, Karlsruhe, Germany) at room temperature (RT) for 15 min. After washing with PBS, the cells were permeabilized with 0.1% Triton-X-100 (solved in PBS) for 15 min at RT. F-actin and the nuclei were stained in SW-13 and H9c2 cells using phalloidin conjugated to Texas-Red (1:400, 40 min, RT, Thermo Fisher Scientific) and 4′,6-diamidino-2-phenylindole (1 µg/mL, 5 min, RT). iPSC-derived cardiomyocytes were incubated overnight with monoclonal mouse anti-α-actinin-2 antibodies (1:200, A7732, Sigma Aldrich, Burlington, VT, USA) in combination with secondary polyclonal goat anti-mouse immunoglobulin G antibodies conjugated with Alexa Fluor 568 (1:200, A11004, Thermo Fisher Scientific).

### 2.5. Confocal Microscopy

Confocal microscopy in combination with deconvolution analysis was performed as previously described in detail using a TCS SP8 system (Leica Microsystems, Wetzlar, Germany) [17]. In brief, three-dimensional stacks were generated and shown as maximum intensity projections using the LAS X software (version 3.5.7.23335) (Leica Microsystems). Deconvolution analysis was performed using the Huygens Essential software (version 23.04.0) (Scientific Volume Imaging B.V., Hilversum, The Netherlands).

### 2.6. Molecular Desmin Model

Recently, the molecular structure of the highly homologous intermediate filament protein vimentin was published [18]. We used this structure to model the desmin anti-parallel tetramer structure using the SWISS-MODELL server (https://swissmodel.expasy.org/ accessed on 10 July 2025) [19]. The tetrameric desmin structure was visualized using PyMOL Molecular Graphics Systems (version 3.1.6.1) (Schrödinger LLC, New York, NY, USA).

### 2.7. Statistical Analysis

Cell transfections were performed in quadruplicate, and filaments or aggregates were manually counted in about one hundred cells. GraphPad Prism 10 software (version 10.6.1) (GraphPad Software, Boston, MA, USA) was used for the generation of pie or bar charts. All data are shown as mean values ± standard deviation. The non-parametric Mann–Whitney test was used for statistical analysis of aggregate and filament formation in transfected cells.

## 3. Results

**Figure 1:**
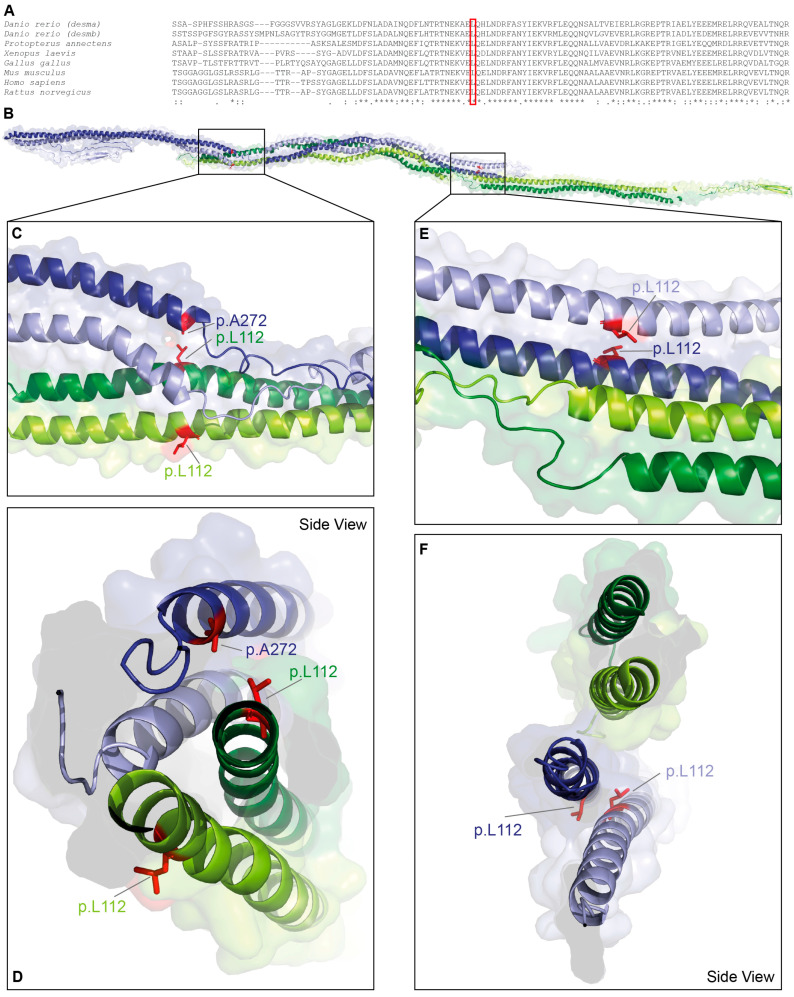
Structural analysis of *DES*-p.L112Q. (**A**) Partial desmin sequence alignments of different vertebrate species. Leucine 112 is highly conserved (highlighted by a red box). (**B**–**F**) Molecular model of the desmin tetramer. The backbone is shown in green, and the leucine 112 residues are shown in red. In addition, alanine 272 is labelled in blue.

*DES*-p.L112Q is localized in a highly conserved stretch at the N-terminus of the 1A domain (Figure 1A). Leucine 112 is a hydrophobic amino acid contributing to the coiled-coil formation between the α-helices of the parallel dimer (Figure 1B–D). Additionally, it mediates presumably hydrophobic interactions with alanine 272 of the antiparallel dimer (Figure 1E,F). Within the antiparallel desmin tetramer, the two constituent dimers are not structurally equivalent. One dimer displays its C-terminal tail domains in a relatively extended, linear orientation, whereas in the opposing dimer, the tail domains appear partially folded back toward the rod domains.

**Figure 2:**
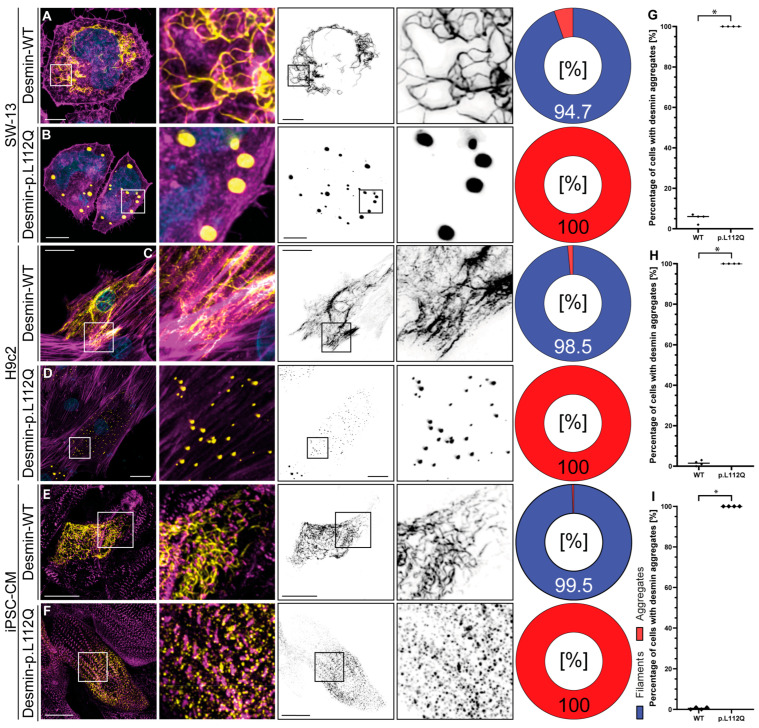
Cellular analysis of *DES*-p.L112Q. Representative cell images of SW-13 (**A**,**B**), H9c2 cells (**C**,**D**), and cardiomyocytes derived from induced pluripotent stem cells (**E**,**F**) are shown. Desmin is shown in yellow or black, F-actin or α-actinin-2 is shown in magenta, and the nuclei are shown in cyan. Scale bars represent 10 µm (SW-13) or 20 µm (H9c2 and iPSC-derived cardiomyocytes). (**G**–**I**) Quantification of the percentage of filament and aggregate-forming cells are shown as pie and bar charts (mean values ± standard deviation). In total, four independent cell transfection experiments (n = 4) were performed, and about 100 transfected cells were analyzed per transfection experiment. The non-parametric Mann–Whitney test was used for statistical analysis (* *p* < 0.05).

Confocal microscopy in combination with deconvolution analysis revealed, independently of the cell type used, a filament assembly defect in desmin-p.L112Q. We used SW-13 cells, since this cell line does not express endogenous desmin or any other cytoplasmic intermediate filament proteins [20]. H9c2 and iPSC-derived cardiomyocytes express, in contrast, endogenous desmin [21,22]. Desmin-p.L112Q aggregates within the cytoplasm (Figure 2), indicating that the hydrophobic interactions within the dimer and tetramer structure of desmin may be disturbed by introducing a polar glutamine residue at position 112. In contrast, wild-type desmin forms filamentous structures of different sizes and shapes in most transfected cells (Figure 2).

## 4. Discussion

Recently, Xing et al. identified the heterozygous de novo variant *DES*-p.L112Q in a pediatric patient with DCM [1]. The authors classified this novel desmin variant according to the guidelines of the American College of Genetics and Genomics (ACMG) [23] as a variant of unknown significance (VUS) fulfilling the PM6, PM2, and PP3 criteria [1]. At the same amino acid position, a different likely pathogenic variant (p.L112R) causes aberrant cytoplasmic desmin aggregation [2]. Comparably, our functional analysis showed a detrimental defect of desmin-p.L112Q similar to desmin-p.L112R. Desmin-p.L112Q forms aberrant cytoplasmic aggregates, indicating an intrinsic desmin filament assembly defect. Recently, we validated the filament assembly assay according the detailed guidelines of the ACMG [24] including twelve different positive and negative controls [17]. Therefore, functional studies indicate an additional strong criterion for the pathogenicity of *DES*-p.L112Q and may be helpful in the classification of genetic variants.

## 5. Limitations

In the future, it may be valuable to characterize further patho-mechanisms using patient-specific iPSC-derived cardiomyocytes from individuals diagnosed with DCM carrying *DES* variants.

## 6. Conclusions

Here, we report that desmin-p.L112Q causes a filament assembly defect similar to other pathogenic *DES* variants. Therefore, our functional data complement the data of Xing et al. [1] and support the re-classification of *DES*-p.L112Q as a likely pathogenic variant rather than a VUS. These findings may be relevant for clinical and genetic counselling of further patients carrying similar *DES* variants.

## Data Availability

All data are contained within the article. The plasmids described are available on request from the corresponding author.

## Abbreviations

ACMG	American College of Medical Genetics and Genomics
DCM	Dilated cardiomyopathy
DMEM	Dulbecco’s Modified Eagle Medium
iPSCs	Induced pluripotent stem cells
PBS	Phosphate-buffered saline
RT	Room temperature
VUS	Variant of Unknown Significance
